# Substance Use Among Older People Living With HIV: Challenges for Health Care Providers

**DOI:** 10.3389/fpubh.2019.00094

**Published:** 2019-04-24

**Authors:** Sherry Deren, Tara Cortes, Victoria Vaughan Dickson, Vincent Guilamo-Ramos, Benjamin H. Han, Stephen Karpiak, Madeline Naegle, Danielle C. Ompad, Bei Wu

**Affiliations:** ^1^Center for Drug Use and HIV Research, College of Global Public Health, New York University, New York, NY, United States; ^2^Rory Meyers College of Nursing, New York University, New York, NY, United States; ^3^Silver School of Social Work, New York University, New York, NY, United States; ^4^Department of Medicine, New York University Langone Health, New York, NY, United States; ^5^Gay Men's Health Crisis, AIDS Community Research Initiative of America Center on HIV and Aging, New York, NY, United States

**Keywords:** aging, HIV, substance use, alcohol, smoking, comorbidities

## Abstract

Older people living with HIV (OPLWH) have higher rates of substance use (tobacco, alcohol, and other drugs) than their HIV-negative peers. Addressing health care needs of OPLWH who use substances is more challenging than for those who do not: they are highly impacted by comorbid conditions, substance use can interact with other medications (including antiretroviral therapy-ART) and reduce their effectiveness, and substance use has been associated with reduced adherence to ART and increased risky behaviors (including sexual risks). People who use substances also suffer disparities along the HIV continuum of care, resulting in lower viral suppression rates and poorer health outcomes. They are especially impacted by stigma and stress, which have implications for HIV treatment and care. Recommendations for health care providers working with OPLWH who use substances include: (1) the need to screen and refer for multiple associated conditions, and (2) training/continuing education to enhance care management and maximize health outcomes.

## Introduction

As treatments for HIV have improved, the population of older people living with HIV (OPLWH) has increased. In 2015 it was estimated that more than 47% of those living with HIV were aged 50 and older, and 7% were 65 and older (1). The proportion of adults living with HIV aged 50 and older increases by an average of 2% annually (2), and 17% of new infections are among people aged 50 and older (1).

Aging and HIV are each associated with a broad range of comorbidities and treatment needs. Addressing the health care needs of OPLWH who use substances, including tobacco, alcohol, and other drugs, is even more challenging than for those who do not use these substances. These challenges come from several sources: OPLWH who use substances are highly impacted by comorbid conditions and substance use can interact with other medications including anti-retroviral therapy (ART) reducing their effectiveness or resulting in unanticipated side-effects. In addition, while some research indicates that OPLWH are more adherent to ART than younger populations living with HIV (3), HIV-infected people who use substances may be less adherent to ART, and suffer disparities all along the continuum of care including lower retention in care, delayed initiation of ART and poorer HIV treatment outcomes (4).

OPLWH have higher rates of substance use than their HIV-negative peers (5). In addition, while rates of substance use largely decline with increasing age in the general population, this has not been found among OPLWH (6). Thus, as the population of those who are HIV-infected continues to age, there is a need for health care providers, especially those working with geriatric populations, to become knowledgeable about the impact of substance use on OPLWH so that their health care needs are optimally identified and addressed.

The authors of this article represent several disciplines, including nursing, psychology, gerontology, social work and epidemiology, and bring multiple perspectives to research and practice. The article will provide an overview of substance use prevalence among OPLWH who are aged 50 and older and many of the comorbidities and associated challenges encountered in managing their health needs. The importance of addressing psychosocial factors and sexual risk behaviors among substance-using OPLWH are also discussed. A final section provides recommendations for screening for the needs of OPLWH and for associated training needs of health care professionals working with them.

## Prevalence of Substance Use Among OPLWH

Surveying 1,000 older adults (aged 50 and older) living with HIV in New York City, the ROAH(Research on Older Adults with HIV) study (7) found that more than half (54%) of the participants were actively enrolled in recovery programs and over one-third reported current (past 3 months) use of illicit substances (37%) or alcohol (38%). Prevalence of specific substances was: tobacco (57%), marijuana (23%), pain killers (38%) cocaine (15%), and heroin (7%) (8). Alcohol is the most frequently used substance among OPLWH (9). Other studies have shown substance use prevalence rates (excluding alcohol) of over 50% in OPLWH (10). Importantly, while injection drug use as a transmission category for HIV has declined in incidence, accounting for 9% of new HIV cases in the US in 2016, it constitutes approximately 18% of people living with HIV (11).

The high rate of substance use among OPLWH is likely to continue. As the baby boomer generation ages, due to their greater experience with mood-altering substances, they use higher levels of alcohol and other substances than previous generations (12).

### Men Who Have Sex With Men

Men who have sex with men (MSM) account for more than half of all people living with HIV (54%) and about 70% of new infections (13). It has been widely documented that MSM are more likely to use illicit drugs as compared to their heterosexual counterparts (14, 15). About one-third of new HIV infections among MSM have been attributed to risk behaviors related to non-injection substance use (including the use of alcohol and other drugs before having sex) (16).

In the ROAH study (7), 53.0% of HIV-positive MSM aged 50 and older in NYC reported alcohol use, 21.4% reported using poppers (i.e., nitrite inhalants), 22.9% reported “hard” drug use (i.e., crystal methamphetamine, cocaine, crack, heroin, ecstasy, GHB, ketamine, and/or LSD/PCP), and 28.0% reported marijuana use. In a more recent study of HIV-positive MSM aged 50 years and older, 48.2% had used an illicit drug in the past 30 days (17).

### Tobacco and Alcohol

Alcohol and tobacco are not always included in studies of substance use, in part because of their legal status. However, because of their widespread use and significant adverse impacts on OPLWH, they are covered in more specific detail below.

#### Tobacco

Smoking is a major risk factor for a variety of life-threatening chronic diseases such as coronary heart disease, diabetes, and cancer (18). Smoking is also a source of inflammation and impaired immune function (19). The health impacts of smoking are of particular concern among people living with HIV, as they are almost twice as likely to smoke than the national average (42.4 vs. 20.6%), are less likely to quit (20) and already have compromised immunity and a predisposition for chronic inflammation (21). Furthermore, adults with HIV who smoke are more likely to be poor, homeless, previously incarcerated, depressed, and have lower educational attainment than those who do not smoke (20), and thus have multiple risk factors for poor health outcomes.

Smoking has been shown to increase the incidence of both AIDS and non-AIDS-related mortality (21, 22). HIV and smoking serve as independent and interacting risk factors for serious conditions such as cardiovascular disease, chronic obstructive pulmonary disease (COPD), human papillomavirus (HPV), and related cancers (21). In addition, smoking may have indirect adverse effects on health by lowering ART adherence (mediated by depressive symptoms) (23), and reducing the effectiveness of ART medication (24, 25). In older adults, smoking may also increase the likelihood of fractures by further decreasing bone mineral density already diminished by the effects of HIV and ART (21, 26).

#### Alcohol

Heavy alcohol use contributes to hypertension, liver disease, cancers, cardiac dysrhythmia, gastrointestinal problems, neurocognitive deficits, bone loss, depression, and impaired judgment leading to risky behaviors (27, 28). Other consequences include suicide, child maltreatment, and unsafe sex and drug injection practices associated with HIV (29, 30). Furthermore, OPLWH are more susceptible to the negative effects of alcohol use, as tolerance lowers with age, metabolism slows, and interaction with prescription medications may impact the effectiveness of medications or exacerbate side effects (31). The prevalence of alcohol use disorders is higher among people living with HIV than the national average (32), and there is higher morbidity and mortality at lower levels of alcohol consumption among HIV-positive individuals compared with those who are HIV-negative (33).

Alcohol has been found to have an impact on HIV disease severity and mortality (9). Heavy alcohol consumption, HIV, and aging independently increase the risk for several comorbidities including cardiovascular disease, liver cirrhosis, diabetes pneumonia and myopathy (34), hypertension and impaired renal function (35). Furthermore, heavy alcohol use among HIV positive people has been linked with reduced engagement at every stage of the HIV care continuum, resulting in increased viral loads, and faster disease progression (36). There is also some conflicting evidence for interactions between alcohol and ART which may reduce ART efficacy, for example by disrupting membrane transporter proteins for some ART medications (37).

Overall, there is a high rate of substance use among OPLWH, and this is related to comorbidities and disease progression. Thus, efforts to screen for substance use and address its consequences are essential in the care of OPLWH.

## Co-Morbidities of OPLWH

OPLWH have a higher number of comorbidities than is found in their non-infected age counterparts (38, 39). In the ROAH study (7), depression was the most common comorbidity, and 40% of participants reported 4 or more comorbidities, with an average of 3.3, about three times higher than seen among community dwelling adults 70 years and older (40). A recent review article (41) noted that among OPLWH, complications associated with aging can appear clinically in essentially every organ system. Vance (42) found that in OPLWH who were >60 years old, 20.5% had coronary artery disease, 67.1% had hypertension, 65.8% had hyperlipidemia, 28.8% had diabetes, 24.7% had peripheral neuropathy, 23.3% had renal disease, 12.7% had hepatitis C, and 38.4% had depression with the success of ART, OPLWH are dying from non-AIDS related conditions more often than AIDS-related conditions (43). Accelerated or accentuated aging has been proposed among OPLWH and attributed as a cause of higher frequencies of co-morbidities (44).

The multimorbidity found among OPLWH has been attributed to several causes, including: (1) the impact of chronic HIV infection (44); (2) behavioral factors related to substance use; (3) elevated rates of inflammation and immune activation (45); and (4) the higher toxicity of early forms of ART (46). Few studies have the power to control for the many variables that contribute to this high rate of multimorbidity in OPLWH (47).

### Polypharmacy and Adherence

The high rates of multimorbidity among OPLWH result in increased pill burden and disease management (42, 48). Polypharmacy has been associated with side effects, drug interaction, and challenges to adherence (49). In one study, OPLWH who were in their 50s were prescribed an average of 12.5 medications (42).

While higher numbers of medications are associated with greater odds of having a fall requiring medical attention for older populations, this is further exacerbated for those with substance use disorders (50). In addition, there are several shared cytochrome enzymes in the metabolism of antiretroviral medications with different substances including alcohol, cocaine, opioids, and marijuana. Therefore, drug-drug interactions between antiretroviral medications and substance use through cytochrome enzymes could decrease the efficacy of antiretroviral therapy, thereby worsening HIV-related outcomes (51).

Many studies have reported relationships between substance use and non-adherence to medications (52) including non-adherence to ART (53). For example, one study of OPLWH examined the relationship between ART non-adherence (number of days when ART was skipped) and days when study participants used alcohol, marijuana, cocaine/crack, opiates, amyl nitrite (poppers), and other drugs. The best adherence (92% reported >90% adherence) was among those reporting no substance use, and the poorest ART adherence was among those reporting polydrug use (<65% reporting >90% adherence) (10).

### Geriatric Conditions

As OPLWH age, there is evidence of an increased burden of age-related chronic conditions (44). These geriatric conditions (or syndromes) have distinguishing features that set them apart from other health conditions—they effect multiple organ systems and are influenced by environmental factors. These conditions are common among vulnerable populations including adults with comorbidities (as is frequently found in OPLWH), and are typically followed by a decline in functional health (54). Common geriatric conditions include falls, cognitive impairment, urinary incontinence, delirium, and frailty (54). The recognition and management of geriatric conditions is critical because they are independently associated with functional decline and mortality (55). When geriatric conditions are addressed, the health status of older adults improves, particularly for vulnerable populations. This is evidenced by decreasing mortality risks, nursing home admissions, and acute care utilization (56, 57). Geriatric conditions are common among OPLWH, including cognitive impairment, falls, urinary incontinence, and difficulty with 1 or more instrumental activities of daily living (58). The recognition and treatment of geriatric conditions in OPLWH should be incorporated into HIV care (59).

Attention to geriatric conditions is particularly relevant for substance-using OPLWH. Problems associated with prolonged substance use and substance use-related comorbidities and behaviors have been linked to accelerated rates of frailty (60, 61). Drug use has a wide array of physiologic effects on the body that may place OPLWH at heightened risk for geriatric conditions. Substance use itself can impact geriatric conditions directly as excess or harmful alcohol use, prescription opioid use, and benzodiazepine use have all been associated with an increase in falls and cognitive changes (60, 62). A recent paper posited the impact of drug addiction on early onset of age-related disease, through toxicity and lifestyle factors (63).

### Cognitive Functioning

Advanced age increases susceptibility to HAND (HIV-associated neurocognitive disorder) in HIV-infected persons, and it is estimated that about 50% of all ART patients present with cognitive impairment (64). Substance use has an impact on CNS structures related to cognitive function. For example, an escalated pattern of cocaine intake is associated with long-lasting damage to the prefrontal cortex, thus affecting cognitive function including executive function, attention, and memory (65). Additionally, stimulant drugs including amphetamine, cocaine, methamphetamine, and ecstasy impact on adult hippocampal neurogenesis. Exposure to stimulant drugs has an effect on neurogenesis regulation in adult brains which can result in drug-induced impairment in cognitive function (66).

Chronic substance use has been shown to lead to impaired cognitive performance. Relationships that have been identified include the following: (1) cocaine affects long-lasting memory (67); (2) amphetamine use is related to attention and impulse control (68); (3) opioids impact attention and performance on memory tasks (69); (4) alcohol affects working memory and attention (70); (5) cannabis impacts cognitive shifting and sustained attention (71); and (6) nicotine has been shown to have an impact on working memory and sustained attention (72).

The manifestations of the cognitive impairments depend on many factors including the types and frequency of substance use, the individual's genetic makeup, age, and environmental factors. The persistence of substance use over time is associated with long-lasting cognitive decline, a particular concern for OPLWH. For example, long-term cannabis users reported having more deficits on memory and sustained attention than short-term users (73). A study of ex-ecstasy abusers found persistent relatively poor score in verbal memory over 2.5 years of abstinence (74). In another study, persons with polysubstance dependence (cocaine or heroin) had deficits in executive function over 5 months of abstinence (75).

The multiple comorbidities found in OPLWH are related to HIV status and aging and impact many organ systems as well as psychiatric conditions such as depression and impairments in cognitive functioning. Thus, care for OPLWH requires a multidisciplinary team and holistic approach.

## Psychosocial Influences on OPLWH Who Use Substances

Psychosocial influences have also been shown to have significant impact on health outcomes of OPLWH. Two important influences on the health of all HIV-infected populations that have been especially prevalent among people who use substances are stigma and stress/trauma.

### Stigma

Stigma has been associated with HIV since early in the epidemic (76) and continues in the post-ART era (77). Emlet (78) noted that OPLWH contend with stigma related to ageism, including social discrimination from their peer networks and employment situations, and institutional/structural discrimination from health care providers.

Older adults who are members of sexual minorities fear rejection and neglect from health care providers, especially non-professional workers. In one study, 83% cited rejection by neighbors in their communities, as well as observations of rejection of partners and friends in long term care (79).

Some of the more recent literature on stigma notes the intersectionality of stigmas (80), i.e., stigma experienced on the basis of multiple characteristics, such as HIV status, older age, racial minority status, gender identity, real or perceived substance use and for MSM, stigma based on their sexuality. For example, intersectional stigma was identified in a study of older Black women living with HIV, who reported stigma related to gender, race, age, and disease (81).

Stigma has been found to be related to poorer health behaviors for PLWH (82, 83), including lower levels of adherence to ART, and lower usage of health and social services. Stigma also impacts disclosure and self-esteem among OPLWH (83). Interventions or factors found to help coping with stigma include social support (80) and spirituality (84).

### Stress and Trauma

Early childhood abuse and PTSD has been associated with subsequent substance use, risky behaviors, and HIV (85, 86). For example, Lee (87) found that among a sample of people who inject drugs, almost one-third (30.9%) reported childhood sexual abuse, and this was associated with syringe sharing. In addition, many people who use substances have experienced poverty, homelessness, or incarceration, with resultant exposure to chronic and acute stressful life events. Minority stress related to sexual orientation has been related to increased substance use (88). Several pathways leading from stress and trauma to substance use and risky behaviors have been proposed (e.g., depression) and trauma-informed therapy for substance users has been recommended (89).

## Risky Sexual Behavior

Many older adults remain sexually active well into their 7th−9th decades of life (90) and OPLWH have been shown to continue to engage in sexual behavior (91, 92). In the ROAH study, 40% of OPLWH reported engaging in anal or vaginal sex in the prior 3 months, and 75% of those sexually active reported having sex more than 2–3 times per month (91). Importantly, almost half of those who were sexually active reported having unprotected anal or vaginal sex in the past 3 months and about 21% of participants reported recent unprotected sex with HIV-negative partners or partners whose HIV status was unknown. Those who were sexually active were more likely to report recent drug use. Further, many studies have shown that substance use is related to risky sexual behaviors, e.g., condomless anal sex among HIV+ MSM (93), and risky sexual behaviors among HIV-infected MSM and women (94). Sexual risk may also vary with partner type. One study of OPLWH found that substance use was related to increased condomless sex with casual partners but not with main partners (95).

Similar to their younger peers, drug use by older adults with HIV is a prime predictor of engagement in high-risk sexual behavior and non-adherence to medications (96, 97). Poor adherence to ART is of concern for the health of the infected patient, and in addition, the resultant high viral loads increase the probability that HIV can be transmitted to sexual partners.

Substance use has been associated with greater sexual risk among MSM, but it is unclear whether sexual risk differs between older and younger MSM (98). In the ROAH Study, erectile dysfunction drugs and poppers were both associated with increased odds for condomless anal/vaginal sex among HIV-positive bisexual and gay men (99). There is some evidence to suggest that cognitive functioning may be an important factor to consider in the association between substance use and sexual risk behavior. In a study of older HIV-positive MSM, drinking to intoxication and current illicit substance use were associated with condomless anal sex when controlling for impairment on two or more cognitive tests, marijuana use, lifetime illicit substance use and HIV diagnosis on or before 1996 (93).

In addition to addressing substance use and co-morbidities of OPLWH, attention to psychosocial factors such as experiences of stigma and stress can also enhance their health status. Sexual health discussions with care providers can serve to reduce risks for both the OPLWH and their sex partners.

## Recommendations for Care Providers

As part of their HIV care, many OPLWH are screened and treated for the co-morbidities identified in this paper, especially non-communicable diseases. However, screening may not be consistent across all patient groups, and as many OPLWH move into geriatric care environments, some important comorbidities, particularly substance use disorders, may be missed. Specific recommendations related to the care of OPLWH who are substance users include patient screening needs and staff training requirements. These are outlined in the following section.

### Screening for Smoking and Providing Cessation Counseling

Many of the risks associated with tobacco use can be reduced by cessation, e.g., bacterial pneumonia in people living with HIV (100). In addition, the time since an HIV-positive individual has abstained from smoking is inversely correlated with risk for cardiovascular disease (101). One study showed that for those over 50 who stop smoking, smoking-related cardiovascular disease risks excess vanishes within 5 years after smoking cessation (102). Smoking cessation is a crucial intervention for promoting positive health outcomes among people living with HIV, especially OPLWH. Multiple interventions have been used for smoking cessation in this population, with varying degrees of success (103). Recommendations for the role of health care providers include the use of a brief intervention which consists of: routinely asking patients about tobacco use and their willingness to stop smoking, efforts to increase motivation to quit, and a range of cessation strategies such as providing nicotine substitution, and referral to stop smoking clinics (102).

### Screen for Alcohol and Other Substance Use

The US Preventative Services Task Forces recommends screening all adults for alcohol misuse (104). The National Institute on Alcohol Abuse and Alcoholism (NIAAA) defines low risk drinking as 1 drink per day (or 7 per week) and no more than 3 drinks per single day for men and women over 65 (105). OPLWH are encouraged to limit or abstain from alcohol consumption. Those with positive screening scores for misuse, hazardous or binge drinking (4 drinks for women and 5 drinks for men in about 2 h) should receive brief behavior counseling. The components of SBIRT (screening, brief intervention, and referral to treatment) provide an integrated approach to screening and intervention (106) and can be undertaken by physicians, nurses, or ancillary care providers (107). A positive score on the screener supports a brief intervention, where information about potential health risks based on the pattern of use is provided. Patients assessed with the likelihood of a moderate to severe substance use disorder should be referred to specialty treatment. This model has yielded positive outcomes across a range of health care settings and patient populations (106). Several screening tools are recommended for use with SBIRT, including computer-assisted self-interview instruments (108).

### Assess for Depression and Other Mental Health Comorbidities

Depression is the most frequent comorbidity among OPLWH (7) and is one of the most underdiagnosed conditions in aging populations (109). Symptoms of clinical depression can be exacerbated in the presence of chronic conditions such as HIV (110). The validity of the Patient Health Questionnaires has been studied in primary care settings and with older adults (111, 112). Screening for stress and trauma should also be considered, as they are associated with substance use.

### Assess for Geriatric Conditions Including Cognitive Deficits

Best practices for addressing geriatric conditions or syndromes is the prevention of geriatric conditions using an interprofessional team knowledgeable in caring for older adults. For example, preventing falls and reducing polypharmacy can prevent the emergence of associated geriatric conditions. Assessment of risk for geriatric conditions, therefore, is a component of integrated care. The Montreal Older Adults Cognitive Assessment (MOCA) is a brief, culturally sensitive tool that has been used extensively with persons with co-occurring chronic medical conditions. It has high sensitivity and specificity for mild cognitive impairment and Alzheimer's dementia (113). Cognitive assessment can provide data to the clinician about the severity of impairment related to NCDs, capacity for psychosocial interaction and the type of health teaching that may be useful in enhancing adherence, reducing risk behaviors, and better health practices generally.

### Screen for Risky Sexual Behavior

Care providers are often uncomfortable discussing sexual intimacy, especially with older populations. The frequency of sexual health discussions between physicians and older patients has been found to be suboptimal (114), perhaps due to discomfort on the part of the health care provider or the assumption that people over the age of 65 are not sexually active. It is important for care providers to ask their patients who are OPLWH (as well as their other older patients) about their sexual behaviors and discuss the need for protection, review condom use skills, and provide condoms. This can also provide patients the opportunity to discuss their questions and concerns about various sexual activities. For OPLWH who are active with HIV-negative or HIV status-unaware sexual partners, PrEP (pre-exposure prophylaxis) should be raised as a prevention tool. Discussion of efforts to end HIV transmission and the campaign U = U (undetectable = untransmittable) can also inform this discussion. Further, exploration of other aspects of sexual health beyond HIV prevention can also be raised for the holistic care of OPLWH.

### Provide Access to Drug Treatment and Harm Reduction Services

There are a wide array of evidence-based treatments available for people who use substances. These include medications for smoking [Chantix, Habitrol, Nicorette (115)], alcohol [naltrexone(Vivitrol), Acamprosate, Disulfiram (116)] and for prescription opioid and heroin use [methadone, buprenorphine and naltrexone (117)]. A variety of behavioral counseling and self-help programs (including 12-step programs), can also be effective independently or often as adjuncts for these medication-assisted treatments.

The use of a harm reduction approach with substance users has increased in acceptance in the health arena, moving the focus of care from abstinence only to reducing the negative consequences of substance abuse. Harm reduction is consistent with the ethical codes for health care providers, which require respect for patients and use of evidence-based care, and has been found to help patients adopt healthy behaviors (118). For OPLWH, harm reduction efforts include assisting patients who wish to access treatment (HIV and substance use) and respecting patients' choices to continue substance use, e.g., for those who engage in drug injection, referring them to syringe exchange programs.

## Training/Continuing Education Needs for Staff in Facilities with OPLWH

As the population of OPLWH grows, there is rising concern that their care providers, and care facilities for them (e.g., assisted living, geriatric care and long-term skilled nursing facilities), are not ready to provide needed services related to substance use. Recommendations for continuing education and training for health care providers treating OPLWH are listed below, and many of these can be incorporated in continuing education and graduate and undergraduate programs for clinicians planning to work with this population. Guidelines for the management of OPLWH are available (www.HIV-Age.org), and should be consulted in developing continuing education programs. Education recommendations include:

### Training Programs on Beliefs About HIV, Sexual Minorities, and Substance Users

Both professional and non-professional care providers may hold stigmatizing attitudes and training should target myths or stigma that may be associated with the OPLWH. Sexual minority men, other persons with non- traditional gender identities, and those who use substances may fear being stigmatized, which can interfere with effective communication. Staff training in such techniques as motivational Interviewing and substance use counseling can also be helpful in understanding the basis for continued substance use (e.g., peer influences and depression), and provide tools for engaging clients.

### Information on Ethical and Legal Obligations to Provide Care, Advocacy, and Support Services (119)

Ethical and legal guidelines for health care providers apply to accommodations in assisted living, nursing and long term care facilities. While facilities are required to post and adhere to Patients' Bill of Rights [e.g., (120)], enhancing staff skills in applying these to advocacy and care delivery may be needed. For example, training on patients' participation in their own care planning is paramount for ethical care delivery.

### Information on Newer HIV-Related Treatments and Care Interventions (e.g., ART Regimens, Pre-exposure Prophylaxis [PrEP] for HIV-Negative Sexual Partners)

As treatment has been centered in HIV/AIDS dedicated centers, providers in care facilities for older populations, not specializing in this area, may need to expand their knowledge in treating this population, including education about new treatment developments.

### Use of Multidisciplinary, Holistic Approaches to Care, Including Patient-Centered Care

These approaches may not be prevalent in long-term care facilities, but continue to be identified as most effective in reorienting health care models and reforming health systems. Appreciation for minority race/ethnic and cultural differences in perceptions of health care services can also serve to enhance patient-centered care.

### Use of a Care Management Model

Comprehensive care with the integration of behavioral and other medical treatment provided by practitioners of multiple disciplines is now advocated in all care settings. Given the complexity of circumstances and comorbidities faced by many substance users, best practice suggests that long term care facilities and ambulatory clinics caring for older adults would be best served by embracing the “care manager” or “case manager” approach to coordinate professional and non-professional providers, and link clients to social services within communities.

### Education on the Management of Co-morbidities

The prevalence of many co- morbidities in this population, with the possibility for interactions of medications, and adverse reactions to prescribed medications, requires training for staff, particularly for managing medications for OPLWH who are using alcohol, cocaine or other substances.

### Education on Evidence-Based Treatment and Best Practices for Substance Use Disorders

The last decade has led to the expanded use of Medication Assisted Treatment (MAT) for opioid treatment, and harm reduction approaches. Practitioners in general health care or geriatric care may be unaware of new biomedical or behavioral treatment models for people who use substances. It has been reported that some nursing facilities refuse to admit patients if they are taking medication to treat opioid addiction (121). Problems emerging around this may be due to gaps in staff training, and persistence in the erroneous belief that 12 step models requiring abstinence, because of their dominance, are the gold standard for addiction treatment. Knowledge of these changes and support for MAT can help OPLWH find suitable program placements (122).

## Limitations and Conclusions

It should be noted that while broad in scope, this paper has several limitations. It was developed to identify key issues that should be considered in providing care to substance -using OPLWH. As such, it does not provide a comprehensive review of the literature. In addition, while some of the recommendations are practiced in many clinical settings (e.g., screening for substance use and referral for drug treatment) they are often not widely implemented across patient groups, especially OPLWH and those entering geriatric care facilities.

As the population of OPLWH continues to increase, clinicians working with those who are substance users will need to address a broad range of issues. For OPLWH, especially those who have been or are currently substance users, care management must go beyond a focus on viral suppression. Integrated care to address their treatment needs, with the contributions of multiple professional disciplines and health care specialties, will be needed to maximize health outcomes for this population. Furthermore, in addition to addressing the individual needs of OPLWH, structural barriers to the delivery of needed health care services must also be addressed, including availability of drug treatment and harm reduction services, as well as social conditions that impact their health (e.g., homelessness).

## Author Contributions

SD conceived of the paper and drafted sections. TC, VD, VG-R, BH, SK, MN, DO, and BW wrote sections of the paper. All authors reviewed the final manuscript.

### Conflict of Interest Statement

The authors declare that the research was conducted in the absence of any commercial or financial relationships that could be construed as a potential conflict of interest.
